# Charges Made by the New Jersey State Board

**Published:** 1900-12

**Authors:** 


### CHARGES MADE BY THE NEW JERSEY STATE BOARD.

CONSIDERABLE space upon another page is given to charges made against Dr. Charles A. Meeker, of Newark, N. J. Ordinarily personal conflicts are not acceptable in this journal. This, however, transcends these narrow limits and becomes of general interest both to the dental profession and other State examining boards. The fact that the daily papers have taken hold of it elevates it to the dimension of a public scandal, and greatly to the injury of Dr. Meeker. We have no comments to make upon this case except to assure Dr. Meeker that he is one of the members of State dental examining boards whose reputation for probity has never been questioned, and in whom the writer has a confidence that frivolous charges fail to disturb.

